# Clinicopathologic Significance of Extranodal Tumor Extension in Colorectal Adenocarcinoma with Regional Lymph Node Metastasis

**DOI:** 10.1155/2016/5620765

**Published:** 2016-04-19

**Authors:** Hyunsung Kim, Abdul Rehman, Yumin Chung, Kijong Yi, Young Chan Wi, Yeseul Kim, Kiseok Jang, Se Min Jang, Seung Sam Paik

**Affiliations:** ^1^Department of Pathology, College of Medicine, Hanyang University, Seoul 04763, Republic of Korea; ^2^Department of Pathology, College of Medicine, Konyang University, Daejeon 35365, Republic of Korea

## Abstract

*Background.* This study investigated the clinicopathologic significance of extranodal tumor extension in colorectal adenocarcinoma with lymph node metastasis.* Method.* Included were 419 patients who underwent curative resection for primary colorectal adenocarcinoma.* Results.* Extranodal tumor extension was observed more frequently in tumors with ulceroinfiltrative gross type (*p* = 0.026), higher histologic grade (*p* = 0.012), high grade tumor budding (*p* = 0.003), vascular invasion (*p* < 0.001), perineural invasion (*p* = 0.015), tumor deposit (*p* < 0.001), high ratio of metastatic/total lymph nodes (*p* < 0.001), and high pN stage (*p* < 0.001). Overall survival was significantly different between an extranodal tumor extension (−) group and an extranodal tumor extension (+) group for both N1 (*p* = 0.022) and N2 homogeneous staging (*p* = 0.007). Both overall (*p* = 0.002) and disease-free survival (*p* = 0.001) were significantly different between the two groups in an N1a homogeneous group and overall survival was significantly different (*p* = 0.016) in an N2b homogeneous group.* Conclusion.* Our study demonstrated that extranodal tumor extension was a useful prognostic factor for colorectal adenocarcinoma with lymph node metastasis, especially in homogeneous pN staging groups.

## 1. Introduction

Colorectal cancer is one of the most common cancers and the second leading cause of cancer-related deaths in the United States [1, 2]. According to cancer statistics, in 2012, South Korea had 28,988 new cases of colorectal cancer: 17,445 (60.2%) in men and 11,543 (39.8%) in women, with a male : female ratio of 1.5 : 1. The crude incidence rate was 57.6 per 100,000 and the crude mortality rate was 16.2 per 100,000 people in South Korea [3].

TNM classification is widely used to evaluate cancer staging and make treatment decisions [4]. Although the TNM staging system has been modified continuously, outcomes of patients with colorectal cancer differ, even among patients with tumors within the same stage [5]. Because colorectal cancer prognosis is still poor, the need for new prognostic factors including histopathological features and molecular subtypes that can stratify patients into different risk group is warranted [5].

The presence of lymph node metastasis and the number of metastatic lymph nodes are useful prognostic factors for colorectal adenocarcinoma [6]. In the 6th American Joint Committee on Cancer (AJCC) TNM staging system, metastatic nodal status was subdivided into N1 and N2 by number of metastatic lymph nodes. In the 7th AJCC TNM staging system, the N category was subdivided more precisely into N1a, N1b, N2a, and N2b within the N1 and N2 categories, according to number of metastatic lymph nodes and presence of pericolonic tumor deposits, which is considered N1c [7]. Extranodal tumor extension (ENTE) to metastatic lymph nodes is widely regarded as a poor prognostic factor for many other solid cancers [8]. A few studies have demonstrated the prognostic value of ENTE in colorectal adenocarcinoma with lymph node metastasis [6, 9, 10]. However, the prognostic impact of ENTE within homogeneous pN staging groups has not been reported.

This study investigated the prognostic significance of ENTE in colorectal cancer with lymph node metastasis. We retrospectively reviewed a consecutive series of 419 patients with colorectal adenocarcinoma who underwent curative resection for primary cancer and evaluated the correlation between ENTE and clinicopathologic factors and investigated survival rates in homogeneous pN staging groups.

## 2. Materials and Methods

### 2.1. Patients and Specimens

**Figure 1:**
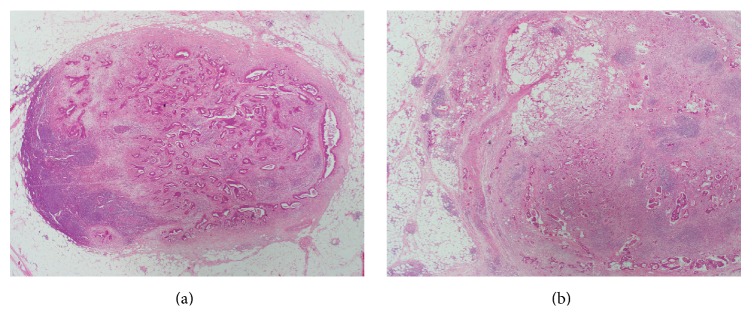
Representative microphotos of lymph node metastasis without extranodal tumor extension (a) and with extranodal tumor extension (b) (hematoxylin and eosin, ×20).

From an institutional database, 419 patients who had undergone curative resection for primary colorectal adenocarcinoma from January 2005 to December 2010 at the Department of Surgery, Hanyang University Hospital, were selected. Excluded were patients who had received neoadjuvant or adjuvant chemotherapy or radiotherapy or those with recurrent colorectal cancer or fewer than 12 lymph nodes. Medical records were reviewed to define clinical characteristics including age, gender, date of surgery, date of last follow-up, date of first local recurrence, and date of first distant metastasis. This study was approved by the Institutional Review Board of the Hanyang University Hospital (HYUH 2015-05-023). Two surgical pathologists (Hyunsung Kim and Seung Sam Paik) reviewed all hematoxylin and eosin stained slides and pathology reports to confirm diagnoses and define clinicopathologic characteristics. TNM staging and other pathologic parameters were determined according to a protocol for examining specimens from patients with primary carcinoma of the colon and rectum [11]. Pathologic data collected included age, gender, tumor size, gross type, tumor location, histologic grade, tumor budding, vascular invasion, perineural invasion, tumor deposit, ratio of metastatic lymph nodes/total lymph nodes (MLN/TLN), pT category, pN category, and status of distant metastasis. All slides of resected lymph nodes were reviewed to determine ENTE. ENTE was defined as a perforation of the nodal capsule by tumor tissue with extranodal growth and evaluated as present or absent [9]. Representative microphotos are in Figure 1. Tumor cell nodule that was discontinuous with the tumor or without surrounding lymphoid tissue was considered a tumor deposit and excluded.

### 2.2. Statistical Analysis

Statistical analysis was performed using SPSS software version 21 (IBM Corp., Armonk, NY, USA). Mann-Whitney *U* and chi-square tests were used to examine associations among ENTE and clinicopathologic parameters of age, gender, tumor size, macroscopic growth pattern, tumor location, histologic grade, tumor budding, presence of lymphovascular invasion or perineural invasion, MLN/TLN ratio, invasion depth (T) category, regional lymph nodes metastasis (N) category, and distant metastasis (M) category. Overall survival rate and disease-free survival rate were determined using the Kaplan-Meier method, and the log-rank test was used to compare groups according to N category. A Cox proportional hazard regression model was used to evaluate the prognostic significance in univariable and multivariable analyses. *p* < 0.05 was considered statistically significant.

## 3. Results

### 3.1. Clinicopathologic Characteristics of Patients

Selected were 419 patients who underwent curative resection for primary colorectal cancer between January 2005 and December 2010. Their clinicopathologic information is in Table 1. Median follow-up was 53 (range: 1–117) months. Mean age at surgery was 63.4 (±11.4) years with 257 (61.3%) men and 162 (38.7%) women with a male : female ratio of 1.59 : 1. Pathological evaluation revealed that 31 tumors (7.4%) were histological grade 1, 196 (46.8%) were grade 2, 168 (40.1%) were grade 3, and 24 (5.7%) were grade 4. By pT stage, 35 (8.4%) were pT1, 42 (10.0%) were pT2, 263 (62.8%) were pT3, 52 (12.4%) were pT4a, and 27 (6.4%) were pT4b. By pN stage, 180 (43.0%) were N0, 48 (11.5%) were N1a, 55 (13.1%) were N1b, 9 (2.1%) were N1c, 62 (14.8%) were N2a, and 65 (15.5%) were N2b. Distant metastasis was identified in 29 (7%) patients. The mean number of removed lymph nodes was 29.2 (±15.78) and of mean metastatic lymph nodes was 3.6 (±6.83).

### 3.2. Correlation among ENTE and Clinicopathologic Parameters

Of the 419 patients, 230 (54.9%) showed lymph node metastasis with 108 (47.0%) with ENTE in metastatic lymph node(s). Correlation among ENTE and clinicopathologic parameters of colorectal adenocarcinoma is in Table 2. ENTE was observed more frequently in tumors with ulceroinfiltrative gross type (*p* = 0.026), higher histologic grade (*p* = 0.012), high grade tumor budding (*p* = 0.003), vascular invasion (*p* < 0.001), perineural invasion (*p* = 0.015), tumor deposit (*p* < 0.001), high MLN/TLN ratio (*p* < 0.001), and high pN stage (*p* < 0.001).

### 3.3. Correlation among ENTE and Overall and Disease-Free Survival in Colorectal Adenocarcinoma

**Figure 2:**
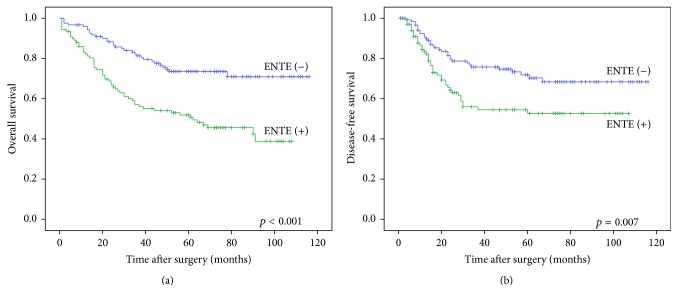
Significant difference between ENTE (−) and ENTE (+) groups in overall survival (a) and disease-free survival (b) (*n* = 230) (Kaplan-Meier method with log-rank test).

The 5-year survival rate was 74% in the ENTE (−) group and 52% in the ENTE (+) group with a significant difference between the ENTE (−) and ENTE (+) groups in both overall and disease-free survival (*p* < 0.001 and *p* = 0.007, log-rank test) (Figures 2(a) and 2(b)). In univariate analyses, ENTE showed a significant effect on both overall survival and disease-free survival (*p* < 0.001 and *p* = 0.009, log-rank test). In multivariate analyses, ENTE was an independent prognostic factor for overall survival (*p* = 0.026, log-rank test). However, ENTE was not an independent prognostic factor for disease-free survival (*p* = 0.234, log-rank test) (Table 3).

### 3.4. Survival Analyses by Homogeneous pN Staging Groups

**Figure 3:**
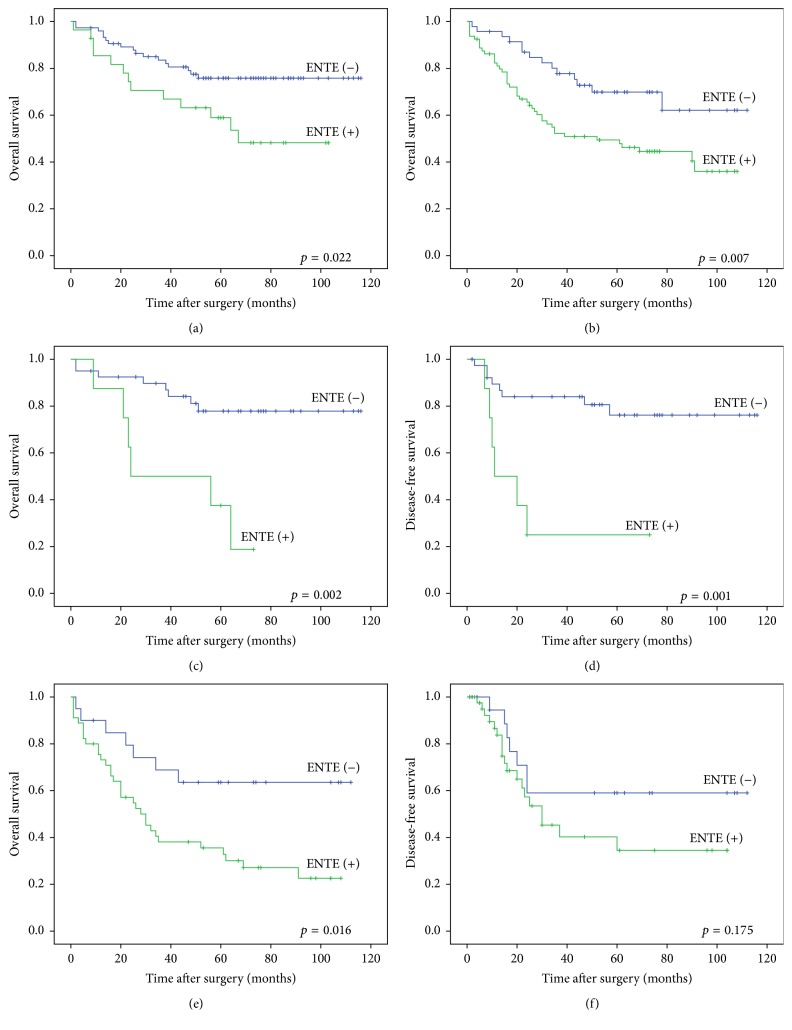
In N1 and N2 homogeneous group analyses, a significant difference was seen between ENTE (−) and ENTE (+) groups in overall survival (a and b). In N1a and N1b homogeneous group analyses, a significant difference was seen in overall survival (c) and disease-free survival (d) by N1a staging group between ENTE (−) and ENTE (+) groups. In N2a and N2b homogeneous group analyses, a significant difference was seen in overall survival (e) of N2b staging group between ENTE (−) and ENTE (+) groups. No significant difference was seen in disease-free survival (f) (Kaplan-Meier method with log-rank test).

To analyze the effect of ENTE in homogeneous pN groups, we grouped the patients with lymph node metastasis into N1 and N2 according to the 6th AJCC staging system and further subdivided them without ENTE and with ENTE. A significant difference was seen in overall survival between the ENTE (−) and ENTE (+) groups in both the N1 (*p* = 0.022) and N2 (*p* = 0.007, log-rank test) homogeneous groups (Figures 3(a) and 3(b)). However, no significant difference was seen in disease-free survival between ENTE (−) and ENTE (+) groups in the N1 (*p* = 0.078) or N2 (*p* = 0.209, log-rank test) homogeneous group (data not shown). We also grouped patients with lymph node metastasis into N1a, N1b, N2a, and N2b according to the 7th AJCC staging system and further subdivided them into without ENTE and with ENTE. The N1a homogeneous group showed significant differences in both overall (*p* = 0.002) and disease-free survival (*p* = 0.001, log-rank test) between ENTE (−) and ENTE (+) groups (Figures 3(c) and 3(d)). However, the N1b homogeneous group showed no significant difference in overall (*p* = 0.453) or disease-free (*p* = 0.958, log-rank test) survival between the ENTE (−) and ENTE (+) groups (data not shown). In the N2a homogeneous group, no significant difference was seen in overall survival (*p* = 0.475) or disease-free survival (*p* = 0.860, log-rank test) between the ENTE (−) and ENTE (+) groups (data not shown). In the N2b homogeneous group, overall survival was significantly different between the ENTE (−) and ENTE (+) groups (*p* = 0.016, log-rank test) (Figure 3(e)). However, disease-free survival was not significantly different between the ENTE (−) and ENTE (+) groups (*p* = 0.175, log-rank test) (Figure 3(f)).

## 4. Discussion

ENTE in metastatic lymph nodes is widely regarded as a poor prognostic factor in many malignancies including gastric cancer [4, 12], bladder cancer [13, 14], papillary thyroid carcinoma [15], penile carcinoma [8], breast cancer [16], non-small cell lung cancer [17], and prostate cancer [18]. Lee et al. and Choi et al. reported that ENTE is associated with advanced T and N stages and is an independent factor for poor prognosis in gastric cancer [4, 12]. Fleischmann et al. and Fajkovic et al. reported that ENTE is an independent prognostic factor for bladder cancer patients with lymph node metastasis, especially with disease recurrence [13, 14]. Distant recurrence is higher in papillary thyroid carcinoma patients with ENTE than in patients without ENTE [15]. Wang et al. found that the degree of ENTE is associated with poor outcome in patients with penile carcinoma [8]. Shigematsu et al. found that ENTE in sentinel lymph node(s) is an independent predictor of nonsentinel lymph node metastasis and of poor prognosis in breast cancer with sentinel lymph node metastasis [16]. Lee et al. showed that ENTE is significantly higher in women with adenocarcinomas of advanced stage, vascular invasion, or p53 expression and that ENTE is associated with poor 5-year survival rate in patients with surgically resected non-small cell lung cancer [17]. Fleischmann et al. reported that ENTE is an indicator lesion for advanced or aggressive prostate tumors with poor outcomes [18].

A few studies have investigated the prognostic value of ENTE in colorectal cancer [6, 9, 10]. Komuta et al. found that ENTE in metastatic lymph node(s) is a poor prognostic factor in a study of 84 patients with colorectal cancer. However, in that study, no significant association was found between ENTE and clinicopathologic parameters and no statistically significant difference was seen in recurrence or survival between pN categories according to the sixth AJCC staging [10]. Yano et al. investigated ENTE in a study of 155 patients with Duke stage C colorectal adenocarcinoma and suggested that ENTE can be a more critical prognostic factor than other parameters such as T status, lymphovascular invasion, and number of positive lymph nodes [9]. Puppa et al. studied pericolonic tumor deposits and extracapsular tumor extension and concluded that the value of ENTE as an independent prognostic factor of stage III cancer is of borderline significance [6].

We found that ENTE was more frequent in cancers with well-known adverse histopathologic features such as higher histologic grade, high grade tumor budding, vascular invasion, perineural invasion, tumor deposit, and pT and pN stages. These results suggested that ENTE was closely associated with aggressive clinical behavior of colorectal adenocarcinoma with lymph node metastasis. In survival analyses, Kaplan-Meier survival curves revealed a significant difference between ENTE (−) and ENTE (+) patients in both overall survival and disease-free survival. These results suggested that ENTE is a poor prognostic factor for colorectal adenocarcinoma with lymph node metastasis.

The prognostic impact of ENTE in homogeneous pN staging groups of colorectal cancer has not been reported. In subgroup analyses by homogeneous pN staging groups, significant differences were seen in overall survival between ENTE (−) and ENTE (+) groups in both N1 and N2 staging groups and overall and disease-free survival in the N1a staging group with and without ENTE. In the N2b homogeneous group, overall survival was significantly different between the ENTE (−) and ENTE (+) groups. These results suggested that ENTE is an important prognostic factor in homogeneous pN staging groups. Therefore, careful pathological examination should be done to detect ENTE in metastatic lymph nodes and pathology reports should specify the presence of ENTE in colorectal adenocarcinoma with lymph node metastasis.

Our study had several limitations in patient selection and definite criteria for ENTE. Neoadjuvant treatment is a well-known effective therapy for colorectal adenocarcinoma that is essential for some patients. However, we could not determine the clinicopathologic correlations between neoadjuvant treatment and ENTE and excluded all patients who received neoadjuvant or adjuvant chemotherapy or radiotherapy. We defined ENTE as perforation of the nodal capsule by tumor tissue with extranodal growth and evaluated ENTE as present or absent. However, this definition cannot be applied in all cases. For example, some lymph nodes have no fibrous tissue covering the lymphoid tissue. Komuta et al. also graded the amount of ENTE by the number of tumor cells in the extranodal space [10]. Definite criteria and grading of ENTE and more studies with a large number of patients are needed.

In conclusion, our study demonstrated that the presence of ENTE was an important prognostic factor in homogeneous pN staging groups. The prognostic effect of ENTE as number of metastatic lymph nodes with ENTE or thickness of ENTE in metastatic lymph nodes should be validated in further studies. We recommend that the number of metastatic lymph nodes and the presence of ENTE be considered in determining the pN stage of colorectal cancer.

## Figures and Tables

**Table 1:** Baseline patient characteristics (*n* = 419).

Factors	Value (%)
Number of patients	419 (100%)
Mean age at surgery (years)	63.4 (±11.4)
Sex	
Male	257 (61.3%)
Female	162 (38.7%)
Histologic grade	
Grade 1	31 (7.4%)
Grade 2	196 (46.8%)
Grade 3	168 (40.1%)
Grade 4	24 (5.7%)
Pathologic characteristics	
pT	
1	35 (8.4%)
2	42 (10.0%)
3	263 (62.8%)
4a	52 (12.4%)
4b	27 (6.4%)
pN	
0	180 (43.0%)
1a	48 (11.5%)
1b	55 (13.1%)
1c	9 (2.1%)
2a	62 (14.8%)
2b	65 (15.5%)
M	
0	390 (93.0%)
1a	25 (6.0%)
1b	4 (1.0%)
Mean number of removed LNs	29.2 (±15.78)
Mean number of metastatic LNs	3.6 (±6.83)

LNs: lymph nodes.

**Table 2:** Correlations between ENTE and clinicopathologic factors in colorectal adenocarcinoma with regional lymph node metastasis (*n* = 230).

Factors	*n*	ENTE		*p* value
		Negative (%)	Positive (%)	
		(*n* = 122)	(*n* = 108)	
Age (year)				0.476^†^
Mean (±SD)	230	62.66 (±10.30)	63.43 (±12.82)	
Gender				0.857^‡^
Male	137	72 (52.6%)	32 (47.4%)	
Female	93	50 (53.8%)	43 (46.2%)	
Tumor size				0.477^†^
Mean (±SD)	230	5.13 (±2.06)	5.33 (±2.12)	
Gross type				0.026^‡^
UI	127	59 (46.5%)	68 (53.5%)	
UF & P	103	63 (61.2%)	40 (38.8%)	
Location				0.275^‡^
Colon	134	67 (50.0%)	67 (50.0%)	
Rectum	96	55 (57.3%)	41 (42.7%)	
Histologic grade				0.012^‡^
Low grade	101	63 (62.4%)	38 (37.6%)	
High grade	129	59 (45.7%)	70 (54.3%)	
Tumor budding				0.003^‡^
Low grade	111	70 (63.1%)	41 (36.9%)	
High grade	119	52 (43.7%)	67 (56.3%)	
Vascular invasion				<0.001^‡^
Absent	158	96 (60.8%)	62 (39.2%)	
Present	72	26 (36.1%)	46 (63.9%)	
Perineural invasion				0.015^‡^
Absent	69	45 (65.2%)	24 (34.8%)	
Present	161	77 (47.8%)	84 (52.2%)	
Tumor deposit				<0.001^‡^
Absent	158	101 (63.9%)	57 (36.1%)	
Present	72	21 (29.2%)	51 (70.8%)	
MLN/TLN				<0.001^†^
Mean ratio (%)	230	15.30 (±14.43)	31.81 (±25.46)	
T stage				0.211^‡^
T1	2	1 (50.0%)	1 (50.0%)	
T2	15	11 (73.3%)	4 (26.7%)	
T3	148	81 (54.7%)	67 (45.3%)	
T4	65	29 (44.6%)	36 (55.4%)	
N stage				<0.001^‡^
N1	103	75 (72.8%)	28 (27.2%)	
N2	127	47 (37.0%)	80 (63.0%)	
Distant metastasis				0.741^‡^
Absent	204	109 (53.4%)	95 (46.6%)	
Present	26	13 (50.0%)	13 (50.0%)	

SD: standard deviation; ^†^Mann-Whitney test; ^‡^chi-square test; UI: ulceroinfiltrative; UF: ulcerofungating; P: polypoid; MLN/TLN: metastatic lymph nodes/total lymph nodes.

**Table 3:** Univariable and multivariable analyses in colorectal adenocarcinoma with regional lymph node metastasis (*n* = 230).

Variable	Univariable analysis		Multivariable analysis	
	HR (95% CI)	*p* value	HR (95% CI)	*p* value
Overall survival				
ENTE (absent versus present)	2.484 (1.603–3.848)	<0.001	1.676 (1.065–2.637)	0.026
Vascular invasion	3.095 (2.034–4.710)	<0.001	2.469 (1.595–3.822)	<0.001
Perineural invasion	1.806 (1.086–3.002)	0.023	1.078 (0.631–1.841)	0.785
Tumor deposit	3.778 (2.477–5.763)	<0.001	2.850 (1.839–4.416)	<0.001
Tumor budding (low grade versus high grade)	1.990 (1.284–3.084)	0.002	1.704 (1.087–2.672)	0.020
Disease-free survival				
ENTE (absent versus present)	1.866 (1.171–2.973)	0.009	1.357 (0.821–2.244)	0.234
Vascular invasion	2.967 (1.858–4.738)	<0.001	1.211 (0.722–2.029)	0.468
Perineural invasion	1.410 (0.834–2.384)	0.200	1.154 (0.651–2.042)	0.624
Tumor deposit	2.205 (1.373–3.542)	<0.001	0.937 (0.556–1.580)	0.807
Tumor budding (low grade versus high grade)	1.244 (0.769–2.012)	0.375	0.888 (0.524–1.503)	0.658

HR: hazard ratio; CI: confidence interval; ENTE: extranodal tumor extension.
